# Adaption and Application of the Four Phase Trials to Traditional Chinese Medicines

**DOI:** 10.1155/2013/128030

**Published:** 2013-08-20

**Authors:** M. Y. Di, J. L. Tang

**Affiliations:** ^1^Division of Epidemiology, The JC School of Public Health and Primary Care, Prince of Wales Hospital, The Chinese University of Hong Kong, Shatin, New Territories, Hong Kong; ^2^Hong Kong Cochrane, Faculty of Medicine, The Chinese University of Hong Kong, Hong Kong

## Abstract

Four phases of trial are widely used in testing drugs, surgery, and diagnosis in Western medicine (WM). The staged testing process helps protect patients from unnecessary harms and control costs while assessing safety and efficacy. In this paper we adapt the four phase trials for traditional Chinese medicine (TCM). As TCM has been used in humans for thousands of years and there has been good preliminary clinical evidence on safety and efficacy for many of its therapies, in most cases its evaluation can start directly in humans, and preclinical laboratory research can be conducted in phase 4 trials after the efficacy is firmly demonstrated. Furthermore, unlike investigational drugs, TCM therapies are various in the certainty of their safety and efficacy and thus should not enter the evaluation process at the same stage. Unlike in WM, clarifying and refining PICO (patients, intervention, comparator, and outcome) are an important part of evaluation of newly designed TCM therapies. The incommensurability between WM and TCM causes additional difficulties in TCM trials regarding defining and choosing PICO, for which some suggestions are made. Observational studies seem to have a greater role in evaluation for TCM although the efficacy must be confirmed with randomized trials.

## 1. Introduction

Medical interventions always consume resources and often have harms or adverse effects, even if they are ineffective. Thus, new medical interventions must be tested rigorously in real practice and in human subjects for their potential efficacy and harms before they are introduced into routine clinical care. The most carefully designed testing and evaluation of medical interventions is the one for new drugs in modern Western medicine, whereas in the long history of medicine, the testing was largely more of a trial and error approach in real clinical practice. Testing of modern drugs follows a staged evaluation process which is normally known as the four phases of trial. This staged approach to testing has also been extended to evaluation of surgical operations and diagnostic technologies [1–6]. In this paper, by describing the concepts of the four phases of trial, and their applications to testing of new drugs, surgical operations, and diagnostic technologies, we hope to identify its relevance and applications to evaluation of traditional Chinese medicine (TCM), which will be followed by a discussion on some special issues in applying the four phase trials to TCM.

## 2. Four Phase Trials in Testing Investigational Drugs in Western Medicine

The phase I–IV trials are referred to the entire process of testing drugs in Western medicine (WM) after it is tested in vitro and in vivo and before it is officially endorsed to be used in routine clinical practice. The four phase trials have been used for decades as the standard approach to evaluating, in human subjects, the safety and efficacy of drugs in WM. This staged testing strategy is primarily proposed for testing (new) substances that are to be used as drugs for treating human patients but have not been thus used in humans before [7–12].

### 2.1. Phase I

It is the first stage of testing an investigational drug in human subjects. Studies of this phase mainly are about safety, tolerability, pharmacokinetics, and pharmacodynamics of the new drug. These issues are usually addressed in a single-armed study in which the drug is administered at subtherapeutic doses in a small group of healthy volunteers (usually 10–100). Vital signs, liver and renal functions, and so forth are closely monitored, for assessing safety. Metabolites of the drug are repeatedly investigated in blood, urine, and so forth. At different time points, to assess the metabolic and pharmacological actions of the drug, which may give more information of the side effects and potential harms of the drug. 

### 2.2. Phase II

Trials of this stage are designed as pilot evaluation of the efficacy. Single-armed studies, quasi-RCTs, sequential trials, crossover trials, and sometimes rigorously planned RCTs may all be used at this stage. Studies often recruit a small or moderate number of target patients (dozens to hundreds) and use surrogate outcomes (such as blood pressure and blood cholesterol). The control group receives either standard treatment or placebo, depending on the availability and efficacy of standard treatments. Dosage and duration of treatment may be modified when deemed necessary in this phase. 

### 2.3. Phase III

 Trials of this stage are the most rigorous testing of the efficacy. Such trials will be conducted only when phase I and phase II trials show promising, favorable results. Bias reduction measures such as random allocation, allocation concealment, and blinding will be used whenever appropriate. A moderate or large sample size of target patients (hundreds to thousands) will be enrolled and endpoint outcomes such as disability and death are used in quantifying the efficacy. Intervention and comparison treatments can be similar to those in the prior stage of testing or more often reflects common real care needs. Such trials are usually conducted in settings where the treatment is normally used rather than in optimal therapeutic environments.

### 2.4. Phase IV

**Figure 1:**
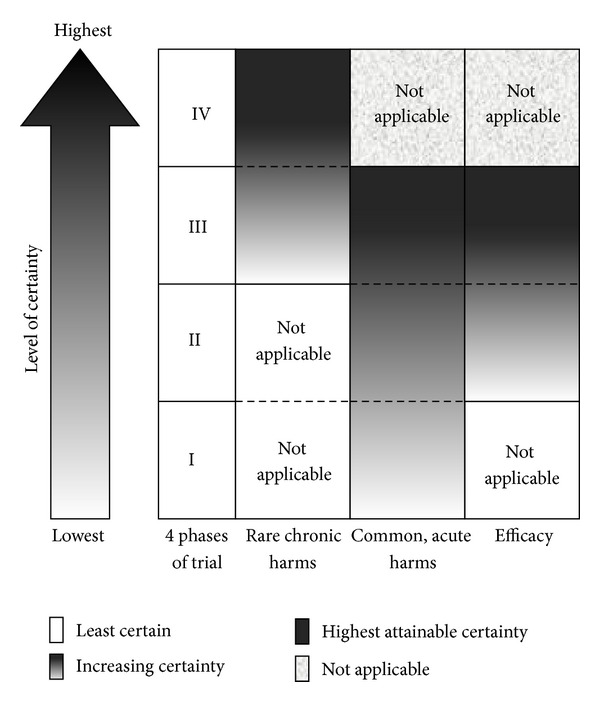
Levels of certainty of efficacy and side effects in different phases of testing.

 Trials of this stage are conducted after a drug has passed phase III evaluation and been authorized into pharmaceutical market for mass production and wide application. As rare chronic harms may take years or decades to occur in a very low frequency, phase III trials are often neither large enough nor long enough to detect such harms. Phase IV trials, often known as postmarket surveillance, are then conducted in which patients who have taken the drug are compared with those who have never taken the drug in order to identify possible rare chronic harms (Figure 1). 

## 3. Why a Staged Approach?

**Figure 2:**
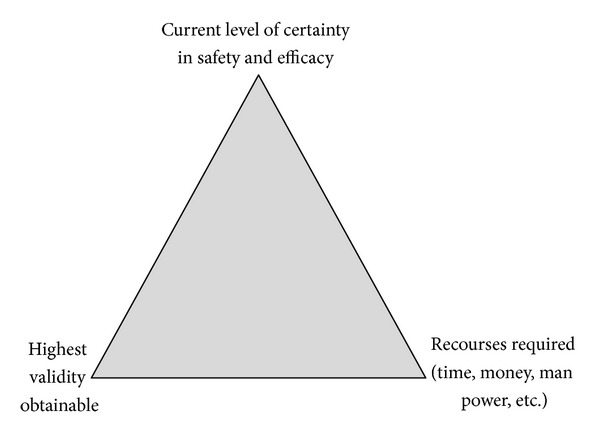
Factors that affect the choice of entry point for testing.

The major reasons for the staged approach to testing new drugs include concerns on efficacy and safety, resources and time, and scientific validity. The ultimate objective of the evaluation is to determine the safety and efficacy with the highest obtainable certainty. The staged testing is particularly designed to protect patients from being harmed and control costs while collecting evidence on safety and efficacy. If evidence on safety and efficacy is not judged sufficiently certain, testing will continue or otherwise stop (Figure 2).

Safety is always the first aspect of concern in the entire evaluation process, as safety is the primary concern for introducing any medical interventions. This principle is rooted in the widely practiced Hippocratic Oath: first, do no harm. Useful treatment must be able to produce more good than harm; drugs that produce harms that are more severe than the treated disease itself are unacceptable. In order to save time and resources in further testing, phase I trials are primarily used to exclude acute adverse effects and harms and describe the drug's pharmacological profiles which may give more information on possible side effects and harms. Testing will go on if the drug is proven lack of severe acute harms.

Pilot efficacy trials (phase II trials) are normally conducted before rigorous, long followup, and resources-consuming trials are launched to confirm the efficacy. In phase II trials, compromises in validity are often made so as to reduce costs and increase efficiency. Intermediate, surrogate outcomes are normally used so as to reduce the time of followup. Trials of compromised quality are often used as well, such as crossover trials and open labeled trials to reduce costs and maximize safety. Trials are often conducted in target patients in normal practice settings with a moderate sample size. A sign of efficacy will take the testing into the most rigorous stage of testing-phase III trial.

Phase III trials can be considered as the ultimate testing for efficacy. Therefore, bias prevention is maximized, which normally include bias-reduction methods such as parallel comparison, randomization, allocation concealment, blinding, and intention to treat analysis. Endpoint outcomes are used and followup period is generally long. Typical patients in average practice settings are recruited. Harms which appear less often can also be detected in such relatively large and long trials.

Phase III trials are however still not large and long enough for detecting rare chronic harms, such as death events which may occur in 10–20 years after treatment and in a frequency of one in a thousand. Discovery of such harms rely largely on postmarket surveillance in which patients who have taken the drug are compared with those who have not to see whether there is any difference in the suspected harmful events. Indeed, many drugs are withdrawn from clinical practice each year due to severe harms found in postmarket surveillance. Of course, rare and chronic are relative. For example, even relatively common acute harms would not be detected in phase III trials on pain-relieving drugs which are normally small and short. 

## 4. Application of Four Phase Trials in Evaluation of Surgical Operations and Diagnostic Methods

More recently, the idea of staged testing is also applied to evaluating surgical procedures [2] and diagnostic methods [5, 13, 14]. However, as operations and diagnostic tests differ from drugs in various aspects, the widely used four phase trials for testing drugs may not be directly applicable in these two areas. Thus, adaptations have been made (Table 1). For instance, unlike chemical substances, most surgical procedures, except the ones implanting synthetic materials, will not lead to toxic effects in the human body. Therefore, phase I trials are mainly about feasibility and safety issues closely related to the procedure itself (such as operational complications), and no pharmacological profiles need to be investigated [6]. Adaptations or compromises are also made in trial design [1]. For example, it is not always ethically acceptable to apply sham surgeries, a key measure to ensure the implementation of blinding [3, 4, 15, 16]. Thus most trials on surgical operations are open-labeled. 

For diagnostic tests, neither they directly introduce toxic substances nor operational complications to the human body, which makes safety scrutiny much less important an issue in phase I studies and in the entire testing. Additionally, the primary aim of a diagnostic method is to distinguish patients with the target disease from those free of the disease. Thus estimation of the sensitivity and specificity rather than safety or efficacy is the primary task in the first two phases of testing. 

As suggested by C. Gluud and L. L. Gluud [5] in Table 1, in phase I trials the diagnostic method is normally used in healthy people to determine the normal range and the cutoff point that can be used for making the diagnosis. In phase II trials, cross-sectional studies rather RCTs are often used to estimate diagnostic accuracy randomized controlled trials may be used in a later stage to evaluate the ultimate benefit of improving health from using the diagnostic methods in real clinical settings.

Alternatively and probably more practical in real practice, a 3-phase testing strategy can be proposed, in which phase II, III, and IV studies are similar to those in Table 1, but phase I is considered generally unnecessary.

The successful adaption of the four-stage testing strategy to surgical operations and diagnostic methods suggests that this staged testing strategy may also be applicable to other areas, such as TCMs. 

## 5. Challenges in Evaluating TCM

TCM is a holistic system of medicine consisting of herbal medicine, food therapy, acupuncture, massage, and therapeutic exercise. It has been practiced in China for thousands of years [17, 18]. However, the efficacy of most TCM therapies has not been firmly scrutinized through rigorous scientific research such as randomized controlled trials. Although the pressure and need for evaluation of TCM has greatly increased in recent years [19, 20], debates about how it should be evaluated continue. The characteristics of TCM which make it different from WM make the evaluation of TCM more complicated and challenging than for WM. Different types of TCM treatments further complicate the situation [21]. Below we will describe some of these features of TCM and discuss their implications for evaluation, with the focus on Chinese herbal medicines (CHM).

First, compared with an investigational drug in WM which is a substance that has never been used in humans or never been used in humans as a drug, Chinese medical herbs and herbal medicines differ in an important way that they have long been used in patients. The long history of clinical use provides good preliminary evidence on safety and efficacy [22]. For example, modern rigorous research confirmed that the widely used Qinghao in TCM could effectively treat malaria with little side effects [23]. 

This feature of CHM has two important implications. First, the basic laboratory research on mechanisms will play much less important a role in evaluating CHM than for drugs in Western medicine. Search for active substances and mechanism of action will doom to fail if a CHM is not clinically efficacious [24]. Second, it would be ethically possible to start the evaluation of CHM in humans as whether we test it or not, CHM will continue to be used in populations in which it is officially recognized such as China [24]. This will dramatically change the strategy for evaluating CHM.

 Second, unlike drugs in WM which do not change in composition and are normally prescribed in a similar dose and duration, CHM is traditionally a highly individualized approach in which herbs prescribed may change over time for the same patient, not to say in different patients [25]. This means in TCM patients will rarely be treated exactly the same way. This individualized approach of CHM will make the RCT inappropriate for evaluating CHM as it can only evaluate one or at most a few fixed treatments at a time [21]. 

Third, the individualized approach of CHM also means that the efficacy of CHM will be determined by the joint work of two factors: the doctor who makes the diagnosis and prescribes and the herbs prescribed. As a result, if a trial fails to show an efficacy, one would not be able to tell whether it is because the doctor was not competent or the herbs did not work [24]. 

Fourth, WM and TCM originated from different world views and are incommensurate with each other [24]. Thus, that they see the same patient differently is like a few blind men trying to find out what an elephant is like. The man who touches the leg thinks the elephant is like a post, while the one who grabs the tail believes it is like a rope. WM may see only the “leg,” whereas traditional Chinese medicine may see only the “tail.” The same disease presents different problems in the two paradigms of medicine. For example, in traditional Chinese medicine, hypertension defined in WM would become a few or many different syndromes in TCM, such as Gan Yang Shang Kang-predominance of the yang of the “liver” [26–28]. Furthermore, in TCM, blood pressure need not be referred to in either making the diagnosis or in judging whether the syndromes have been improved or deteriorated. The incommensurability between WM and TCM will make the disease, patient, diagnosis, and prognosis in a trial incomprehensible to WM physicians and the lay public.

Fifth, it is difficult to design placebos for CHMs. The distinctive features of a CHM in appearance, smell, and taste are difficult to mimic by substances other than herbs [29, 30]. If using other different herbs to make the placebo, it would be difficult to rule out any beneficial or harmful effects of the placebo herbs [17]. In addition, the changing appearance of herbal decoctions over time makes preparation of placeboes more difficult [31].

Last but not least. CHMs differ in certainty of their safety and efficacy. Many classical formulas are still widely used. New formulas of current herbs are also “designed” and prescribed to patients everyday in clinical practice [32, 33]. New forms or routes of delivery have been invented, such as solutions for *iv* injection [34–38]. New herbs or substitutes to traditional herbs and in particular to medical materials of animal sources may be discovered or invented [39]. These treatments are various in the degree of certainty of their safety and efficacy. This is to say that different CHMs may have a different need for evaluation and should not be treated the same way. Importantly, different CHMs should probably have a different starting point in the evaluation process. In principle, in order to protect patients and save resources, the entry point for a CHM should depend on the degree of certainty we currently have on its safety and efficacy (Figure 2). Thus, an important issue in evaluating CHMs is to assess the current level of certainty of safety and efficacy of treatments. 

## 6. CHMs Vary in Certainty of Their Safety and Efficacy 

**Figure 3:**
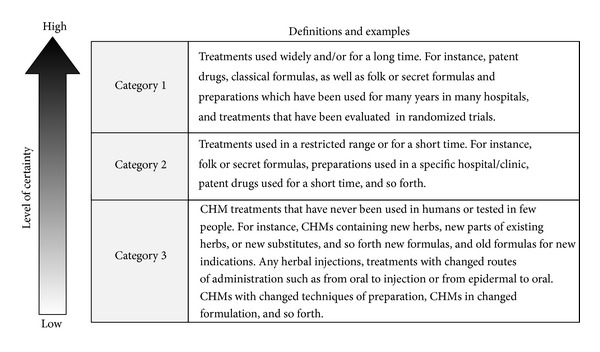
Classification of current Chinese herbal treatments according to the level of certainty of their safety, efficacy, and/or PICO.

The level of certainty in this context is a subjective matter and so far little effort has been made regarding it. It is reasonable to assume that the level of certainty of the safety and efficacy of a CHM treatment is positively related to how long and how widely the treatment had been used. This is to say that the greater the wideness and history of use is, the more certain it will be for safety and efficacy. Based on this assumption, we can divide CHM therapies into three categories according to the level of certainty (Figure 3). 

For CHMs in category 1 we have the highest certainty in safety and efficacy. Typically they would include those that have been used for decades or hundreds of years and still nationally or very widely used in China or other countries. Many patent drugs [40, 41] and classical formulas will fall into this group. We suggest including in category 1 also new CHM formulas that have already been evaluated in randomized controlled trials. For these treatments, the patient, dosage, duration of treatment and outcomes for benefits, and outcomes for side effects and harms are relatively clear.

For CHMs in category 3 we have least certainty of their safety and efficacy. Typically they would include treatments using new herbs or new substitutes, injections, more risky routes of delivery (e.g., from epidermal to oral or form oral to *iv* injection), formulas for newly discovered diseases, and old formulas for different diseases. They normally have not been used in humans at all or just tested in a few cases. For these therapies, there remains a lot of uncertainty about the target patient, dosage, duration of treatment and outcomes for benefits, and outcomes for side effects and harms. We also suggest including in this category therapies for which many doctors have justifiable doubts about their safety. 

Category 2 would include all the rest that cannot be classified into category 1 or 3. That means we have a moderate certainty in safety and efficacy for these treatments.

Having said that we admit that the classification is not based on hard evidence and is suggested only as a general rule for starting the testing process. Because of its subjectivity, we also suggest lowering the category by one grade when there is any doubt or concern. 

## 7. New Four Phase Trials for Staged Testing of CHMs 

**Figure 4:**
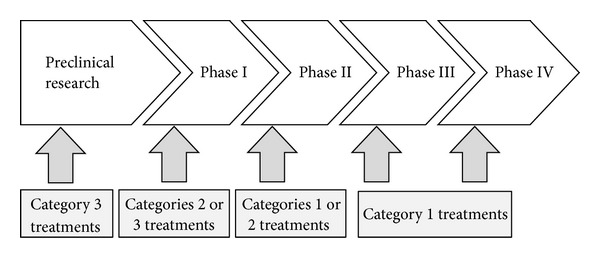
Suggested entry points at the staged testing system for different categories of Chinese herbal treatments.

Given the characteristics of TCM and varying certainty of safety and efficacy of the therapies described previously, there is a need to slightly redefine the objectives of four phase trials for testing drugs to make them suitable for the evaluation of CHM. As the objectives change, trial designs and PICO at each phase need also be adjusted.  Figure 4 presents the new four phase trials we propose to be used in evaluating TCM. The most important difference from the phase four trials for testing drugs is that different CHMs enter different phase of trial according to the degree of certainty of their safety and efficacy and use PICO deemed appropriate (Figure 4).  Table 2 summarizes the objectives and choice of PICOS (S for trial design) in our strategy for testing of CHM.

### 7.1. Phase I

 Trials of this stage for CHMs focus on safety evaluation and finding suitable PICO for the treatment. By finding suitable PICO, we mean to find out what patients to treat according to diagnosis, severity and demographic factors, what specific herbs to use, what dosage range, how long to treat, what outcomes to use to quantify benefits, and what outcomes to use to detect side effects and hams. 

For instance, patients' characteristics favoring greater benefits can be determined in case series. Different dosages and durations could be examined in *n* of 1 trials and possibly crossover trials. As many CHMs have been used in clinical practice, we emphasize the important role of using routinely collected data in phase I trials in TCM. Multiple regression analyses of routine data can be conducted to determine the PICOS. 

Routine laboratory testing such as blood counts, blood chemistry, renal and liver functions, and ECG, should always be conducted in phase I trials to detect any acute side-effects and harms. We believe that no or no thorough pharmacological studies similar to Western drugs would be necessary at this stage of testing for CHMs.

Many treatments in category 2 and those in category 3 with explicit animal research evidence on toxicity can be directly submitted to phase I trials for testing of their safety in humans. Clarifying and refining PICO are a unique feature in testing CHM. 

### 7.2. Phase II

 Trials of this stage are for initial evaluation of efficacy and further refining of PICO. Phase II trials can also help detect possible side effects and harms that are relatively less acute and frequent. Many treatments in category 1, some in category 2 with good evidence on safety and relatively clear definition of PICO, and those that successfully passed phase I trials can be directly submitted to phase II trials as pilot testing for efficacy and/or refining of PICO. 

### 7.3. Phase III

 Trials of this stage are for confirming efficacy and acute and frequent side effects and harms. Many treatments in category 1, some in category 2, and those that successfully passed phase II trials [41] can be submitted to final efficacy testing. We suggest subjecting these CMHs directly to phase III trials not because there is sufficient certainty that they are effective but because most of them are pretty safe and have been and will continue to be used in human patients unless phase III trials can exclude those that are unlikely to be effective. 

### 7.4. Phase IV

Trials of this stage are for surveillance on rare, chronic side effects and harms. Some treatments in category 1 and those that passed phase III trials can be submitted to phase IV trials. We suggest basic lab research on active ingredients for action and mechanisms be conducted only after efficacy is firmly approved [24]. 

## 8. Some Methodological Difficulties 

In this section, we discuss a few methodological issues in designing phase III trials of CHM as an example to demonstrate the methodological difficulties and possible solutions in evaluating CHM by using clinical trials. These issues are mostly related to the choice of PICO rather than bias-reduction methods such as randomization.

### 8.1. Patients

 WM and TCM see the same patient differently and make different diagnoses. Should patients of a WM disease or of a TCM disease be used? TCM is supposed to work most effectively if it is used to treat patients defined within its own system [42]. If patients of the same TCM diagnosis are recruited, many different diseases defined in WM will be included in the same trial and the disease and result will be inexplicable to WM doctors and patients. Conversely, if patients of the same WM diagnosis are recruited, many different diseases defined in TCM and many different treatments would have to be included in the same trial. If an efficacy is shown, it would be difficult or impossible to tell which treatments work and in whom they work. A compromised approach is to recruit patients with the same WM disease and then group and treat patients according to TCM diagnoses [42, 43]. This is a commonly used approach.

### 8.2. Intervention

TCM advocates individualized treatment but the same trial requires the same treatment. The completely individualized approach is in theory most desirable to show the efficacy but least desirable in promoting its use in particular in non-Chinese patients. Completely standardized treatments are the opposite in strength and weakness. A compromised approach is often used in which patients of the same WM disease are divided into 2-3 groups according to their TCM diagnoses and treated differently [44–46]. 

### 8.3. Control

 Placebo treatment is important for successfully maintaining blinding. Unlike with drugs, it is difficult to design a good placebo treatment for herbal formulas. A few ideas have been proposed, while no ideal method can be recommended. It is probably most difficult to design a placebo treatment for herbal decoctions, the commonest form of CHM treatment. Like in WM, it is suggested to use the tested treatment in a very low concentration (say, 1/10–1/30 of the treatment dose) and with additional flavorings and colors added to mimic the smell, taste, and color of the tested therapy. 

For example, edible pigments have been used to produce the color and cereals to generate sediments in placebo decoctions [17, 31]. Studies showed that for placebo made from 20-fold diluted experimental decoction, baked rice was a successful additive [31]. The validity of a placebo treatment can be evaluated before it is used in real trials. Placebo quality checklist is a widely used tool for assessing the degree to which the placebo can successfully blind doctors and patients [47]. However, it is also advisable in placebo-blind CHM trials to conduct a built-in study to evaluate the actual successfulness of blinding at the end of the trial.

### 8.4. Outcomes

 Both outcomes defined in WM and those in TCM can be used in CHM trials. It is argued that trials are likely to draw the same conclusion about the efficacy regardless the outcomes used. First, outcomes on patients' feelings, such as pain [48] and itching, are the same in both TCM and WM. Second, outcomes in both TCM and WM are correlates of different aspects of the same underlying disease and should show improvement if the underlying disease is effectively controlled or in particular cured. 

For example, both blood pressure and syndrome pattern of hyperactivity of liver-YANG (gan yang shang kang) in hypertension are different aspects of the same underlying disease. If hypertension is effectively controlled, improvement should be demonstrated in both. It is also argued that improvement in any WM outcomes or TCM outcomes should be seen as a sign that the treatment is working. As TCM outcomes normally appear more quickly than WM outcomes, they can be used to monitor the treatment in phase III trials or as surrogate outcomes in phase I and II trials. 

### 8.5. Other Issues

As TCM treatment is more like surgery than medicine [6], the efficacy is determined by both the physician and the herbs used. Thus, competent TCM physicians and quality herbs [49] should be used in pilot efficacy trials. In addition, PICO should be described in plain language rather than TCM language if the study report is aimed at Western medicine doctors and the public. This provides an additional challenge for trials in TCM.

## 9. The Role of Real World Studies in Evaluating TCM

It has been heatedly debated in particular in China that randomized controlled trials are done in patients and settings that differ from real practice and thus cannot answer the question whether the treatment truly works in reality [50, 51]. This problem is particularly severe for TCM given the problems of the RCT when applied to TCM. The extreme argument that in real practice patients are treated according to their needs rather than determined by chance (i.e., by randomization) tends to totally decline the relevance of RCTs in evaluating medical interventions [52]. Real world studies (RWS) in which researchers only observe has been proposed and promoted as the solution to the RCT. Big data collected in routine clinical practice readily for use and powerful statistical methods available for analysis seem to have made the RWS particularly attractive over RCTs [53, 54]. Many symposiums and conferences have been organized in China to develop RWS in particularly in the TCM field (such as the Third CORE Summit and the Twelfth National Conference of Clinical Epidemiology). 

However, the debate about the possible difference in the effect of interventions between testing environments and real practice is not new. The term “efficacy” was coined to reflect the former and effectiveness the later. Large randomized controlled trials in which average patients with a certain disease are all invited, allocated, and treated in usual care settings are particularly designed to address the issue of effectiveness [55]. Indeed, there is still a difference even between large trials and real practice as treatment is not initiated by chance in reality. But is RWS a replacement or complementary for the RCT?

In clinical evaluation of interventions, research methods can be largely divided into observational and experimental. The dividing factor between them is randomization, with which the study is an experiment or otherwise an observation. Randomization also makes other bias reduction methods meaningful and possible in a clinical trial; these methods include allocation concealment, blinding, high follow-up rate, and intention to treat analysis. As a result, randomization makes the RCT much more powerful for control of confounding and other biases than observational studies. RWS excludes randomization, it becomes observational in nature. Observational studies are more prone to biases than RCTs. Thus, RCTs provide more trustworthy evidence on the effects of medical interventions than real world observational studies. 

In evaluating investigational drugs, the so-called real world observational studies have an important role in the early stage of testing on safety and acute harms and also in investigating rare chronic harms at the last stage (Table 2) but the RCT must be used to confirm the efficacy as evidence from RWS is not sufficiently reliable [51]. RWS evidence on effectiveness is meaningful only when the RCT has shown a positive result but RWS failed to confirm it. This difference cannot be used to prove the effectiveness shown from RCTs is wrong but reveal a phenomenon that the treatment benefit is heterogeneous and is more effective in some circumstances than others or more likely that the treatment requires service of certain standard so as to be able to deliver any beneficial effects. 

However, RWS seem indeed to have a great role in the evaluation of TCM in particular when big clinical data are available. Routinely collected data can be used to identify possible efficacy, side effects, and harms for TCM therapies that have been widely used in patients [52]. However, such studies are observational in design and cannot be used as final proof for efficacy although observation of harms that are more severe than the disease itself can be used as final evidence for stopping the use of the related treatment. 

## Figures and Tables

**Table 1:** Main objectives of each stage of testing in human subjects in the process of evaluating new drugs, surgical procedures, or diagnostic methods of Western medicine.

	Phase I	Phase II	Phase III	Phase IV
Drugs	(1) Assessment of acute side effects (2) Investigation on metabolic and pharmacological profiles	Pilot efficacy evaluation	Confirmation of efficacy	Surveillance on rare chronic harms and effectiveness in practical settings

Surgical procedures	(1) Safety assessment (mainly operational complications) (2) Feasibility assessment	Pilot efficacy evaluation	Confirmation of efficacy	Surveillance on chronic safety and long term effectiveness in practical settings

Diagnostic methods	Determination of normal range and initial diagnostic cutoff point	Evaluation of specificity and sensitivity	Evaluation of clinical consequences after introducing the test	Surveillance on long term clinical consequences in practical settings

**Table 2:** Main objectives and PICOS (Patient, Intervention, Comparator, Outcome, and Study design) of trials in different stages of testing in TCM.

	Phase I	Phase II	Phase III	Phase IV
Objectives	(1) General safety evaluation (2) PICO specification	Pilot testing on efficacy and on acute, common side effects and harms	Full-scale testing on efficacy and on acute, common side effects	(1) Surveillance on rare chronic harms (2) Investigation on mechanisms and active substances

Study designs	(1) Case series, *n* of 1 trials (2) Analysis of routine data (3) Observational studies (4) Non-/quasi-RCTs, cross-over trials sequential trials, and so forth	(1) Analysis of routine data, observational studies (2) Non-/quasi-, open-labeled, cross-over trials, sequential trials, small-scale RCTs, and so forth	Rigorously-designed RCTs, large multicentre RCTs	(1) Analysis of routine data (2) Observational studies (cohort studies and case control studies) (3) Laboratory studies on mechanisms and active substances

Patients	(1) People with or without the target WM disease (2) Sample size: dozens	(1) Patients with the target WM disease and specific characteristics (2) Sample size: dozens to hundreds	(1) More representative patients (2) Sample size: hundreds to thousands	(1) Usually those who have received the treatment and those who have not (2) Sample size: thousands and often more

Interventions	CHMs with varying herb components, doses, routes, therapeutic durations, and so forth	CHM of relatively fixed combination of herbs, dose, route, and so forth, which are determined in phase I	CHM similar to that in phase II or more fixed	CHM similar to that in phase II and III possibly with minor modifications in real practice

Controls	No control, or no treatment, standard proven effective treatments or placebo as control	No treatment, or different doses or delivery routes, standard proven effective treatments, or placebo as control	Standard proven effective treatment or placebo	Usually those patients who have not taken the treatment

Outcomes	Routine laboratory tests (e.g., blood counts, renal and liver functions, and ECG) for detecting side effects and harms, and surrogate outcomes defined in WM/TCM for detecting benefits	Surrogate outcomes for beneficial effects and appropriate outcomes for acute common harms	Endpoint outcomes for benefits and appropriate outcomes for acute common harms	Endpoint outcomes for rare chronic harms such as deaths
